# Construction and validation of an innovative prognostic nomogram for overall survival in cervical cancer patients with lung metastasis: an analysis utilizing the SEER database

**DOI:** 10.3389/fonc.2024.1397454

**Published:** 2024-05-08

**Authors:** Linlin Chang, Kangkang Zhao

**Affiliations:** ^1^ Department of 2st Gynecologic Oncology, Jilin Cancer Hospital, Changchun, China; ^2^ Department of 4st Radiotherapy, Jilin Cancer Hospital, Changchun, China

**Keywords:** cervical cancer, lung metastasis, nomogram, overall survival, SEER

## Abstract

**Purpose:**

To facilitate patient consultation and assist in clinical decision-making, we developed a predictive model to analyze the overall survival (OS) rate of cervical cancer patients with concurrent lung metastasis for 6 months, 1 year, or 2 years.

**Methods:**

We extracted data on patients diagnosed with cervical cancer and concurrent lung metastasis between 2010 and 2020 from the Surveillance, Epidemiology, and End Results (SEER) database. Through a random assignment process, these patients were allocated to either a training cohort or a validation cohort, maintaining a 7:3 ratio. Utilizing both univariate and multivariate Cox regression analyses, we determined the independent prognostic factors influencing OS. To enhance predictive accuracy, we developed a nomogram model incorporating these identified independent prognostic variables. Model effectiveness was subsequently assessed using various metrics, including receiver operating characteristic (ROC) curves, calibration plots, and decision curve analysis (DCA).

**Results:**

We gathered data on 1330 patients diagnosed with cervical cancer with lung metastases. An OS nomogram was developed, accounting for factors such as histological type, presence of metastases in other organs (brain, liver), surgical interventions, radiation therapy, and chemotherapy. The ROC curves, calibration plots, and DCA curves demonstrated the commendable predictive performance of the nomogram in assessing the prognosis of cervical cancer patients with lung metastases in both the training and validation cohorts.

**Conclusion:**

By utilizing clinical data from the SEER database, we have effectively devised a nomogram capable of predicting the 6-month, 1-year, and 2-year survival rates of cervical cancer patients with lung metastases. The nomogram boasts high accuracy, offering precise prognostic predictions. Its implementation can guide the formulation of individualized follow-up and treatment plans for enhanced patient care.

## 1 Introduction

Cervical cancer is the most prevalent malignant neoplasm of the female reproductive system. According to Globle Cancer Statistics 2020, there were 604,127 new cases of cervical cancer globally, resulting in 341831 deaths. Both the incidence and mortality rates rank fourth among malignant tumors affecting women (1). According to the latest statistics, the incidence and mortality of cervical cancer have been steadily increasing, particularly among young women in China (2). Upon initial diagnosis, approximately 13% of patients present with tumors that have already metastasized to nearby or distant organs. Research indicates that the 5-year survival rate for cervical cancer patients without metastasis is 91.5%. However, at the onset of metastasis, the 5-year survival rate decreases to 16.5%. Notably, the occurrence rate of cervical cancer with lung metastasis ranges from 2.2% to 9.1%, with patients exhibiting concomitant lung metastasis experiencing significantly decreased survival rates (3, 4). Hence, a comprehensive examination of the pathological features and prognostic factors associated with lung metastasis in cervical cancer patients represents a pivotal concern within the realm of clinical treatment.

Currently, research on patients who are diagnosed with cervical cancer lung metastasis at the time of initial diagnosis, both domestically and internationally, is predominantly characterized by small sample studies or individual case reports. There is limited exploration of factors influencing the survival of these patients, and the commonly employed TNM staging system has limitations in accurately discerning individual survival disparities. Moreover, among patients classified under the same stage, survival rates demonstrate heterogeneity. Therefore, the predictive capacity of the FIGO staging system, commonly used for prognostication, is not comprehensive and demonstrates a need for improved accuracy (5, 6). Consequently, the construction of an accurate and effective prognostic model for cervical cancer patients with lung metastasis holds paramount clinical importance. Nomograms, as tools for assessing disease risk and prognosis, have gained widespread application in clinical practice. Nomograms streamline a multitude of intricate factors into a unified numerical model, facilitating the prediction of event probabilities. In recent years, miRNAs have emerged as crucial tools in the predictive analysis and management of cancer. Thus, utilizing large and reliable datasets from the Surveillance, Epidemiology, and End Results (SEER) database, establishing a nomogram to predict the overall survival (OS) of patients with cervical cancer and lung metastasis and evaluating its predictive accuracy will contribute to guiding clinical treatment decisions and prognostic assessments.

## 2 Materials and methods

### 2.1 Data source and patient selection

We sourced data from the SEER database, a National Cancer Institute-supported repository, utilizing SEER Stat software (version 8.4.3; Incidence—SEER Research Data, 17 Registries, Nov 2022 Sub (2020–2022 varying)) (http://www.seer.cancer.gov/seerstat). The SEER database, which has been regularly updated since 1973, encompasses cancer diagnosis and survival information for approximately 30% of the U.S. population. Access to all SEER data is freely available with publicly accessible ethics approval.

**Figure 1:**
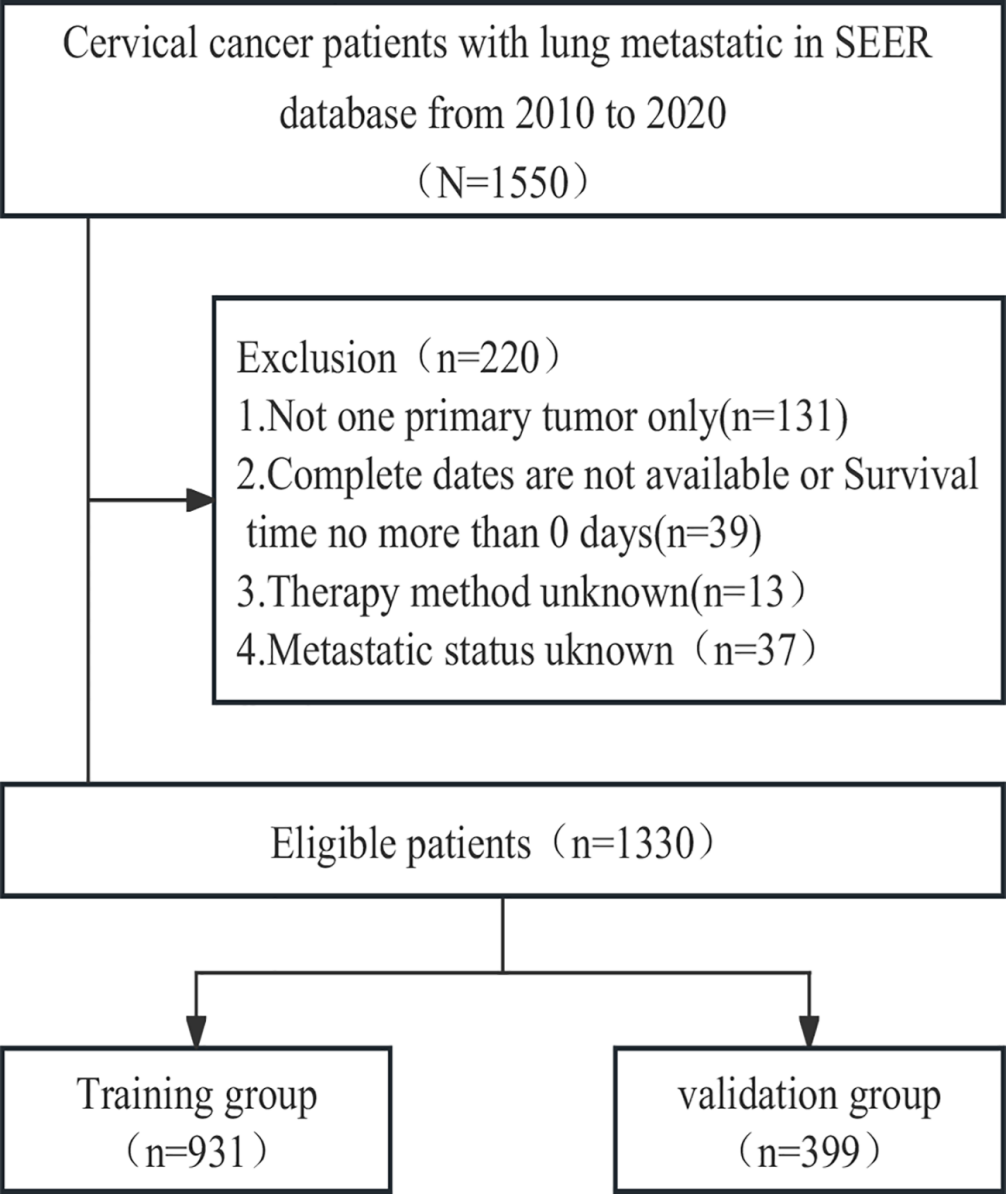
Flowchart of patient selection.

To identify patients with malignant cervical cancer, we applied specific criteria, including the primary site labelled C53.0-C53.1 and C53.8-C53.9 according to the Site and Morphology Primary Site, the behavior recoding for analysis labelled ‘Malignant,’ and the extent of disease SEER combined sets at DX-lung labelled ‘Yes.’ Given that information on distant metastasis sites was first collected in the SEER database in 2010, we restricted the year of diagnosis to the period between 2010 and 2020. The selection criteria and research process are illustrated in **Figure 1**.

### 2.2 Clinical variables and outcomes

**Figure 2:**
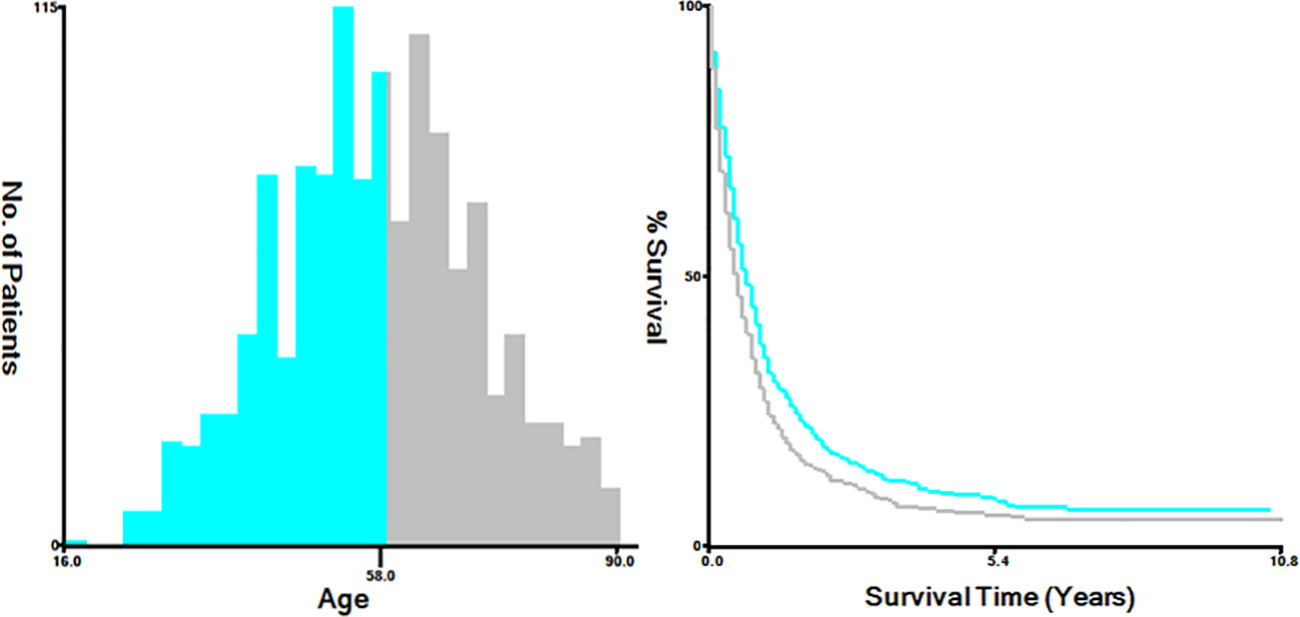
The optimal cutoff values for age were 58.

The variables extracted from the SEER database included diverse factors, including the year of diagnosis, age at diagnosis (categorized as ≤58 and >58 years), race (classified as white, black, and others, incorporating American Indian/Alaskan Native, Asian or Pacific Islander), marital status (grouped into married, single, which includes divorced, separated, widowed, never married or domestic partner, and unknown), primary site (endocervix, exocervix, overlapping lesion of cervix uteri, and cervix uteri), histology (such as squamous cell carcinoma, adenocarcinoma, and others), grade (Grade I, Grade II, Grade III/IV, and unknown), T stage (T1, T2, T3, T4, and Tx), N stage (N0, the N1, and Nx), metastatic site (including the bone, brain, and liver), and treatment method, involving chemotherapy, radiation, and surgery of the primary tumor. Additionally, survival months and vital signs were considered. For analytical purposes, we utilized X-tile bioinformatics software (Yale University, USA, Version 3.6.1) to categorize patients according to age into two groups: ≤58 years and >58 years (**Figure 2**) (7).

Our main measure of interest was OS, defined as the period from the time of cervical cancer diagnosis to the time of the last follow-up or the time of death from any cause.

### 2.3 Statistical analyses

All patients included in the study were randomly allocated to training or validation cohorts at a ratio of 7:3. This randomization was achieved utilizing the “create Data Partition” function within the R “caret” package to ensure the even distribution of outcome events. The training cohort was utilized for the development of a nomogram, while the validation cohort served to validate the model. Categorical variables are presented as percentages and were compared using the Chi-square test. Survival curves were generated using the Kaplan–Meier method, and the log-rank test was applied for analysis. Univariate and multivariate Cox regressions were conducted to discern the significance of variables concerning OS. In the training cohort, the covariates incorporated into the multivariate Cox proportional hazards models were determined through a backwards stepwise method based on the smallest Akaike information criterion (AIC) value. This approach was intended to identify variables that contributed minimally to the loss of prognostic information (8, 9).

Nomograms predicting 0.5-, 1-, and 2-year OS were developed utilizing independent prognostic factors. The discriminatory capacity of the nomogram was evaluated using 0.5-, 1-, and 2-year time-dependent area under the curve (AUC) values. An AUC ranging from 0.5 to 1 indicates the discriminative ability of the nomogram—higher values suggest superior discrimination. An AUC exceeding 0.7 is indicative of excellent discriminative performance.

To gauge the precision of point estimates from the nomogram-predicted survival against actual survival, calibration curves were generated. The bootstrapping method, involving 500 resamples, was applied to produce calibration curves for validating the nomogram in both the training and validation cohorts. Additionally, the net benefit was computed through decision curve analysis (DCA), which provided insights into the ability of the nomogram to predict clinical outcomes (10).

This report follows the guidelines outlined in the Strengthening the Reporting of Observational Studies in Epidemiology (STROBE) network (11). All analyses and graphical representations were executed using R software version 4.3.2 (www.r-project.org). All tests were two-sided, and a significance level of P < 0.05 was considered indicative of statistical significance.

## 3 Results

### 3.1 Baseline characteristics of the study population

Between 2010 and 2020, a cohort of 1550 cervical cancer patients with lung metastasis from the SEER database was initially identified. Through a meticulous selection process, 1330 eligible patients were randomly assigned to either the training cohort (N=931) or the validation cohort (N=399). Throughout the study period, the overall follow-up duration ranged from 0 to 130 months, with a median follow-up time of 6 months. In both the training and validation cohorts, the follow-up times ranged from 0 to 130 months and 0 to 117 months, respectively, with a corresponding median follow-up time of 6 months. Following the cutoff date for follow-up, a total of 1113 patients died, and remarkably, only 49 patients (3.6%) experienced mortality unrelated to cervical cancer. The detailed baseline characteristics of the patients in both the training and validation cohorts are presented in **Table 1**.

**Table 1:** Patient characteristics of the training cohort and the validation cohort.

Variables	Training cohort, n (%) (n = 931)	Validation cohort, n (%) (n = 399)	Overall, n (%) (n = 1330)	P value
Year of diagnosis				0.688
2010–2014	377 (40.5)	167 (41.9)	544 (40.9)	
2015–2020	554 (59.5)	232 (58.1)	786 (59.1)	
Age				0.541
≤ 58	493 (53)	204 (51.1)	697 (52.4)	
> 58	438 (47)	195 (48.9)	633 (47.6)	
Race				0.179
White	678 (72.8)	271 (67.9)	949 (71.3)	
Black	160 (17.2)	79 (19.8)	239 (18)	
Other	93 (10)	49 (12.3)	142 (10.7)	
Marital status				0.496
Married	318 (34.2)	147 (36.8)	465 (35)	
Single	570 (61.2)	238 (59.6)	808 (60.7)	
Unknown	43 (4.6)	14 (3.5)	57 (4.3)	
Primary site				0.471
Endocervix	105 (11.3)	41 (10.3)	146 (11)	
Exocervix	9 (1)	7 (1.8)	16 (1.2)	
Overlapping lesion	15 (1.6)	9 (2.3)	24 (1.8)	
Cervix uteri	802 (86.1)	342 (85.7)	1441 (86)	
T stage				0.505
T1	88 (9.5)	42 (10.5)	130 (9.8)	
T2	160 (17.2)	62 (15.5)	222 (16.7)	
T3	334 (35.9)	145 (36.3)	479 (36)	
T4	125 (13.4)	65 (16.3)	190 (14.3)	
TX	224 (24.1)	85 (21.3)	309 (23.2)	
N stage				0.956
N0	248 (26.6)	103 (25.8)	351 (26.4)	
N1	541 (58.1)	235 (58.9)	776 (58.3)	
NX	142 (15.3)	61 (15.3)	203 (15.3)	
Histology				0.469
Squamous cell carcinoma	615 (66.1)	266 (66.7)	881 (66.2)	
Adenocarcinoma	186 (20)	70 (17.5)	256 (19.3)	
Other	130 (14)	63 (15.8)	193 (14.5)	
Grade				0.165
I	19 (2)	7 (1.8)	26 (1.9)	
II	169 (18.2)	54 (13.5)	223 (16.8)	
III/IV	405 (43.5)	176 (44.1)	581 (43.7)	
Unknown	338 (36.3)	162 (40.6)	500 (37.6)	
Bone metastatic				0.432
No	691 (74.2)	305 (76.4)	996 (74.9)	
Yes	240 (25.8)	94 (23.6)	334 (25.1)	
Brain metastatic				0.052
No	881 (94.6)	388 (97.2)	1269 (95.4)	
Yes	50 (5.4)	11 (2.8)	61 (4.6)	
Liver metastatic				0.889
No	702 (75.4)	303 (75.9)	1005 (75.6)	
Yes	229 (24.6)	96 (24.1)	325 (24.4)	
Surgery				0.107
No	849 (91.2)	375 (94)	1224 (92)	
Yes	82 (8.8)	24 (6)	106 (8)	
Radiation				0.624
No	461 (49.5)	191 (47.9)	652 (49)	
Yes	470 (50.5)	208 (52.1)	678 (51)	
Chemotherapy				0.571
No	327 (35.1)	133 (33.3)	460 (34.6)	
Yes	604 (64.9)	266 (66.7)	870 (65.4)	
Status				0.118
Alive	159 (17.1)	58 (14.5)	217 (16.3)	
Dead	772 (82.9)	341 (85.4)	1113 (83.7)	

T, Tumor; N, Lymph Node.

The demographic and clinicopathologic profiles of the patient cohort revealed that a predominant proportion were white (71.3%), unmarried or single (60.7%), diagnosed with squamous cell carcinoma (66.2%), and exhibited Grade III/IV disease (43.7%). The cervix uteri was identified as the primary site in the majority of patients (86%). Among the 1330 patients, 25.1% presented with bone metastases, 4.6% with brain metastases, and 24.4% with liver metastases. Surgical interventions were conducted in 106 (8%) women; 51% of the patients underwent radiotherapy, and 65.4% received chemotherapy. Notably, there were no significant differences in demographics or clinicopathologic characteristics between the training and validation cohorts (all P > 0.05).

### 3.2 Construction of the nomogram

**Figure 3:**
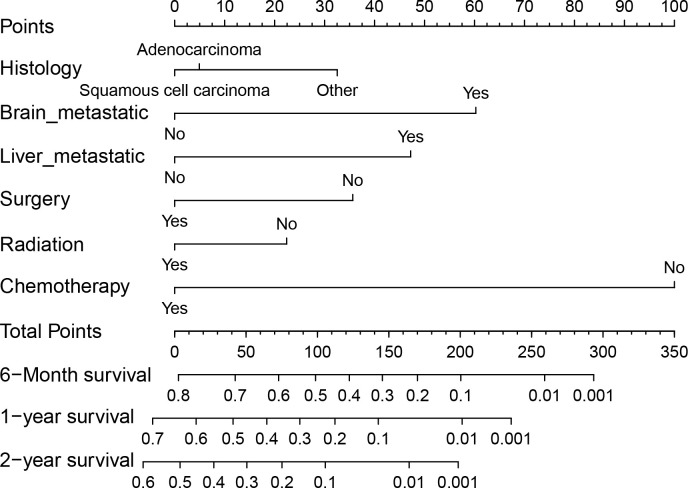
Nomogram for predicting 0.5-, 1- and 2-year OS for patients with cervical cancer and lung metastases in the training cohort.

We conducted both univariate and multivariate Cox regression analyses within the training cohort to ascertain the prognostic importance of the variables under consideration. In the univariate analysis, factors such as age at diagnosis, primary site, histology, presence of lymph node metastases, receipt of surgical treatment, radiotherapy, chemotherapy, and the presence of brain, liver, and bone metastases were found to be associated with OS (P<0.05, as indicated in **Table 2**). These significant variables were subsequently included in the multivariate analysis. Multivariate analysis revealed that histology, surgical treatment, radiotherapy, chemotherapy, brain metastasis, and liver metastasis were independent prognostic factors for OS among cervical cancer patients with lung metastases (P<0.05, as shown in **Table 2**). Drawing from these six independent risk factors identified through multivariate analysis, a nomogram was constructed to predict the 0.5-, 1-, and 2-year OS (**Figure 3**).

**Table 2:** Univariate and multivariate analyses of the prognostic factors for OS in the training cohort.

Variables	Univariate analysis		Multivariate analysis	
	Hazard ratio (95% CI)	P value	Hazard ratio (95% CI)	P value
Year of diagnosis				
2010–2014	Reference			
2015–2020	0.972 [0.842–1.122]	0.699		
Age				
≤ 58	Reference		Reference	
> 58	1.206 [1.046–1.389]	0.01	1.028 [0.886–1.192]	0.72
Race				
White	Reference			
Black	1.078 [0.895–1.299]	0.426		
Other	0.849 [0.664–1.086]	0.193		
Marital status				
Married	Reference			
Single	1.148 [0.987–1.335]	0.074		
Unknown	1.054 [0.737–1.508]	0.772		
Primary site				
Endocervix	Reference		Reference	
Exocervix	0.729 [0.295–1.799]	0.492	0.679 [0.272–1.693]	0.406
Overlapping lesion	1.501 [0.835–2.697]	0.174	1.415 [0.778–2.573]	0.255
Cervix uteri	1.283 [1.019–1.616]	0.034	1.156 [0.899–1.488]	0.259
Histology				
Squamous cell carcinoma	Reference		Reference	
Adenocarcinoma	0.939 [0.784–1.124]	0.495	1.093 [0.896–1.334]	0.381
Other	1.340 [1.091–1.646]	0.005	1.484 [1.196–1.843]	< 0.001
Grade				
I	Reference			
II	0.865 [0.514–1.456]	0.586		
III/IV	1.250 [0.757–2.063]	0.384		
Unknown	1.387 [0.838–2.296]	0.204		
T stage				
T1	Reference			
T2	0.890 [0.663–1.195]	0.437		
T3	1.036 [0.794–1.352]	0.794		
T4	1.229 [0.906–1.667]	0.185		
TX	1.241 [0.937–1.642]	0.132		
N stage				
N0	Reference		Reference	
N1	1.103 [0.936–1.301]	0.243	1.157 [0.977–1.369]	0.091
NX	1.407 [1.122–1.765]	0.003	1.132 [0.897–1.430]	0.297
Surgery				
No	Reference		Reference	
Yes	0.566 [0.433–0.740]	< 0.001	0.680 [0.514–0.900]	0.007
Radiation				
No	Reference		Reference	
Yes	0.630 [0.547–0.726]	< 0.001	0.769 [0.661–0.895]	0.001
Chemotherapy				
No	Reference		Reference	
Yes	0.287 [0.247–0.334]	< 0.001	0.308 [0.262–0.361]	< 0.001
Brain metastatic				
No	Reference		Reference	
Yes	1.863 [1.381–2.513]	< 0.001	2.028 [1.494–2.754]	< 0.001
Liver metastatic				
No	Reference		Reference	
Yes	1.751 [1.490–2.058]	< 0.001	1.727 [1.459–2.045]	< 0.001
Bone metastatic				
No	Reference		Reference	
Yes	1.203 [1.024–1.414]	0.025	1.034 [0.871–1.227]	0.704

T, Tumor; N, Lymph Node.

### 3.3 Validation of the nomogram

**Figure 4:**
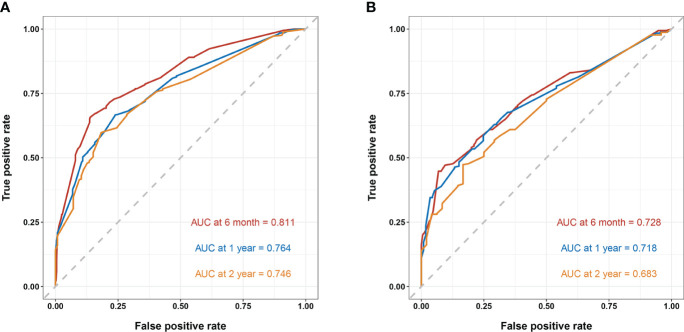
**(A)** nomogram ROC curves to predict 0.5-, 1-, and 2-year OS in the training cohort; **(B)** nomogram ROC curves to predict 0.5-, 1-, and 2-year OS in the validation cohort.

**Figure 5:**
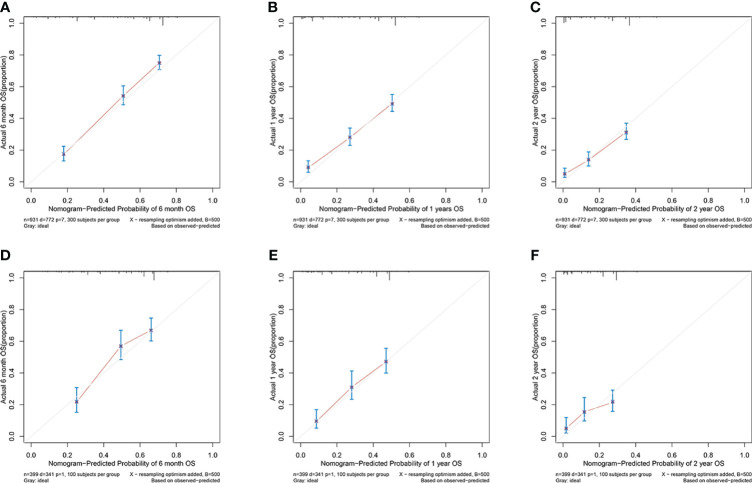
**(A–C)** nomogram calibration plots to predict 0.5-, 1-, and 2-year OS in the training cohort; **(D–F)** nomogram calibration plots to predict 0.5-, 1-, and 2-year OS in the validation cohort.

**Figure 6:**
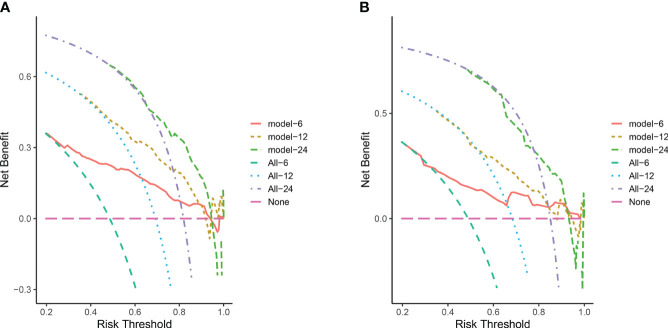
**(A)** DCA for predicting 6-, 12-, and 24-month OS in the training cohort; **(B)** DCA for predicting 6-, 12-, and 24-month OS in the validation cohort. The pink horizontal line represents that all patients in the model die, and there is no clinical benefit. The light green line, blue line and purple line represent the survival of all patients corresponding to 6, 12, and 24 month respectively, and the curve between the 2 represents the benefit of decision-making.

In the training cohort, the area under the curve (AUC) values for the time-dependent receiver operating characteristic (ROC) curves were 0.811, 0.764, and 0.746 for 0.5-, 1-, and 2-year overall survival OS, respectively. In the validation cohort, these AUC values were 0.728, 0.718, and 0.683, respectively (**Figure 4**). These findings indicate the consistent and robust discriminatory ability of our nomogram. The calibration plots, which assessed the concordance between the nomogram predictions and the actual observations for the 0.5-, 1-, and 2-year OS in both the training and validation cohorts, exhibited favorable consistency (**Figure 5**). Furthermore, the decision curves depicted improved clinical applicability for predicting the overall survival of cervical cancer patients with lung metastases (**Figure 6**).

## 4 Discussion

Considering the long-term survival of patients with cancer metastasis, it is important to clarify the prognostic factors of patients and develop reliable survival prediction models. Compared with traditional prediction tools, nomograms, as visualization tools for predicting the risk of disease occurrence and prognosis assessment, have been applied to evaluate the prognosis of patients with distant metastasis of multiple cancers, and all have demonstrated good predictive value (5, 12–15). Currently, there is a notable absence of dependable models for predicting the survival outcomes of individuals diagnosed with cervical cancer and lung metastasis, both domestically and internationally. To address this gap, our study developed a nomogram leveraging data from the SEER database. The nomogram’s performance was rigorously assessed using metrics such as the ROC curve, area under the curve, calibration plot, and DCA. Our findings collectively demonstrate that our nomogram exhibits robust discriminatory ability and accurate predictive capabilities and is of practical value. The SEER database, managed by the National Cancer Institute (NCI), is a comprehensive epidemiological repository providing invaluable insights for research in the field. The SEER database contains extensive information on cancer patients across the United States, including patient demographic characteristics, tumor diagnosis, treatment, and survival data. Due to its large sample size and long-term tracking characteristics, the SEER database has important research value in the field of cancer research. Analysis using this database will have important advantages and credibility.

Studies on the prognostic factors of lung metastasis in cervical cancer patients are the focus of clinical research, and there are obvious differences in the research results. Pulmonary metastases are secondary diseases of hematogenous dissemination and usually present as single or multiple nodules (16, 17). With the strengthening of cancer patients’ medical awareness and the continuous improvement of hospital follow-up technology, very small metastases can be detected earlier, markedly improving the detection rate of lung metastasis. Because most cervical cancer patients with lung metastases usually do not experience symptoms associated with lung metastases, it is impossible to treat metastatic lesions in a timely manner. Cervical cancer patients with lung metastasis exhibit favorable outcomes under three specific conditions: 1) absence of metastasis to other organs beyond the lung, regardless of lymph node involvement; 2) presence of ipsilateral lung metastasis; and 3) ≤4 lung metastases (18).From the analysis it is evident that patients who received surgery or radiotherapy to the primary tumor had a better prognosis. This would suggest that patients who developed lung metastases after locoregional treatment have a better prognosis than those presenting with *de novo* metastases.

The influence of pathological type on the prognosis of cervical cancer patients is still controversial. Some researchers believe that the pathological type of adenocarcinoma is an independent risk factor for the prognosis of cervical cancer patients with lung metastasis and is also a risk factor for lung metastasis of cervical cancer (19). Studies have shown that histopathological type, age, isolated lung metastasis, tumor size, and lymph node metastasis are prognostic factors affecting survival after lung metastasis in patients with cervical cancer. Patients with 3 or 4 lung metastases had lower 5-year survival rates than those with 1 or 2 lung metastases (42.2% vs. 0%, P = 0.0003) (20). Cox multivariate regression analysis revealed that histology, surgical intervention, radiotherapy, chemotherapy, brain metastases, and liver metastases were independent risk factors influencing overall survival OS in cervical cancer patients with lung metastasis. This finding is also in general agreement with previous studies.

This study showed that the survival period of patients with cervical cancer with lung metastasis was relatively short, so the treatment of metastatic cervical cancer is a major clinical difficulty. The nomogram showed that chemotherapy is the best choice for stage IVB patients with lung metastasis of primary cervical cancer without surgical indications. This finding is consistent with those of previous studies. The rapid development of targeted therapy and immunotherapy has revolutionized the treatment of many cancers. There have also been breakthroughs in the development of targeted drugs, including antiangiogenic drugs, tyrosinase inhibitors, and epidermal growth factor receptor blockers. The National Comprehensive Cancer Network (NCCN) guidelines recommend bevacizumab combined with systemic chemotherapy as the standard treatment for patients with distant metastasis of cervical cancer (21). Clinical studies have demonstrated that cisplatin plus paclitaxel with bevacizumab or tolopotecan plus paclitaxel with bevacizumab is effective in treating stage cervical cancer patients and extends their survival (22, 23). Bevacizumab combined with chemotherapy improves the survival rate of patients with recurrent or metastatic cervical cancer and is listed as the first-line therapy for recurrent, metastatic cervical cancer (24, 25). With the increasing use of immune and targeted therapies for treating patients with cervical cancer with lung metastasis, incorporating relevant data into prognostic nomograms will become more accurate and useful.

Nonetheless, it is important to acknowledge certain limitations in this study. First, as the study was retrospective, there was potential for selection bias during the patient selection process. Additionally, due to the limited clinical information available in the SEER database, several valuable clinical factors were not considered in the analysis, such as the absence of tumor markers, HPV infection status, imaging data, and details regarding the treatment of pulmonary metastases, the details of the cycles and doses of chemotherapy could not be obtained. Furthermore, the database primarily comprised Caucasian patients, necessitating external validation and adjustment of the model in diverse populations to ensure its generalizability. Finally, external validation was not performed due to current constraints in experimental conditions. To mitigate this limitation, we adopted a 7:3 ratio for study population allocation, with 30% earmarked for internal validation. The robustness demonstrated by the internal validation results supports the reliability of the model.

Based on the SEER database, this study identified independent prognostic factors for patients with cervical cancer lung metastasis and successfully constructed a survival prediction model for such patients which has good accuracy and clinical application value. Future studies will continue to expand the sample size, include multicenter native patients for validation, and explore the inclusion of other potential predictors to further improve the prediction accuracy and generalizability of the model.

## 5 Conclusion

In summary, our study effectively devised a nomogram capable of predicting the 6-month, 1-year, and 2-year survival rates of cervical cancer patients with lung metastases utilizing clinical data from the SEER database. This nomogram, constructed during our research, demonstrates notable advantages, shows high accuracy and has substantial clinical application value.

## Data availability statement

The original contributions presented in the study are included in the article/supplementary material. Further inquiries can be directed to the corresponding author.

## Ethics statement

Ethical consent was waived due to the SEER database contains anonymous patient information. All data from the SEER database were open access.

## Author contributions

LC: Writing – original draft. KZ: Writing – review & editing.
